# Long-Term Effects of Sinus Floor Elevation with and Without Bone Graft: A Systematic Analysis of Randomized Clinical Trials

**DOI:** 10.3390/medicina61010104

**Published:** 2025-01-13

**Authors:** Andrei Paul Tent, Ioan Andrei Țig, Simion Bran, Andra Zlotu, Alexandru Mester, Florin Onisor

**Affiliations:** 1 Department of Oral and Maxillo-Facial Surgery, Faculty of Medicine and Pharmacy, University of Oradea, 410087 Oradea, Romania; tent_andrei@yahoo.com; 2 Department of Dental Medicine, Faculty of Medicine and Pharmacy, University of Oradea, 410028 Oradea, Romania; itig@uoradea.ro; 3 Department of Maxillofacial Surgery and Implantology, University of Medicine and Pharmacy “Iuliu Hatieganu”, 400012 Cluj-Napoca, Romania; dr_brans@yahoo.com (S.B.); florin.onisor@umfcluj.ro (F.O.); 4 Department of Oral Health, University of Medicine and Pharmacy “Iuliu Hatieganu”, 400012 Cluj-Napoca, Romania; andrazlotu@gmail.com

**Keywords:** sinus lift, bone graft, dental implant, clinical trial

## Abstract

*Background and Objective:* The aim of this systematic review was to assess clinical trials on the efficiency of sinus lift techniques with and without bone grafting in the atrophic posterior maxilla. *Materials and Methods:* This article was written under the PRISMA and the Cochrane Handbook for Systematic Reviews of Interventions guidelines. PubMed, Scopus, and Web of Science databases were electronically searched until December 2023. The risk of bias was assessed according to Cochrane Risk of Bias tool guidelines. Statistical analysis was performed for implant survival rate (ISR), marginal bone loss (MBL), and endo-sinus bone gain (ESBG). *Results:* At the end of the electronic search, 5 clinical trials were considered eligible. Statistical analysis was achieved for osteotome sinus floor elevation. The ISR at 3 years had a risk ratio (RR) of 0.98 [0.90, 1.07] (CI 95%), *p* = 0.7, and at 5 years, RR 1.02 [0.93, 1.11] (CI 95%), *p* = 0.68. The MBL, at 3 years, indicated a weighted mean difference (WMD) of 0.01 [−0.15, 0.16] (CI 95%), *p* = 0.93, and at 5 years, WMD of −0.08 [−0.53, 0.37] (CI 95%), *p* = 0.73. ESBG at 3 years had a WMD of −0.44 [−1.05, 0.17] (CI 95%), *p* = 0.16, and at 5 years, WMD of −0.61 [−1.63, 0.41] (CI 95%), *p* = 0.24. *Conclusions:* The available evidence underlines that the osteotome sinus floor elevation technique without bone graft may be used.

## 1. Introduction

The invention of dental implants was revolutionary, offering patients a second chance at fixed teeth with all their functional and aesthetic benefits [1]. Past studies claim that to ensure good osseointegration of the implants, a residual bone height (RBH) of 8–10 mm is necessary [2]. In the conditions of the pneumatization of the maxillary sinuses, this ideal RBH is rarely reached in the molar region [1,2]. To avoid the complications that could arise secondary to the perforation of the sinus membrane, a series of sinus lift techniques have been developed over time [3]. The approach to the sinus floor can be performed transcrestally or laterally with or without the addition of bone grafts [4]. Although there is a wide variety of bone grafting choices, there is currently no consensus on the ideal material, each having its advantages and disadvantages that must be taken into consideration in each patient [5]. It is well known that the autologous bone graft has the best osteoinductive and osteogenetic properties but has the drawback of donor site morbidity [6]. Complications at the level of the donor site vary depending on the location, the size of the graft, the surgical technique, and the associated pathology of the patient [1,2,3,4,5,6]. In order to avoid the donor site’s possible complications, different authors started to use different types of biomaterials mixed or not with platelet-rich fibrin (PRF) [7].

Other authors prefer the use of allografts [8]. However, their non-approval in certain countries restricts their use on a considerable scale [9]. Additionally, the possibility existing in the past of contracting certain viral diseases, makes some patients and surgeons alike to be reluctant to accept them as a bone grafting choice [9]. However, due to the development and improvement of various sterilization techniques for these types of grafts, the risk of contamination of a form of infection is almost nil these days [9].

From the desire to remove these shortcomings and also to decrease the rate of complications and lower the costs and surgical time, a series of studies have been undertaken to evaluate if sinus floor elevation without bone graft can be successful for implant placement [10,11]. These studies have brought to light the fact that the blood clot formed between the sinus membrane and the sinus bone wall has osteoregenerative properties [12]. The sinus membrane must not be perforated during the surgery in order to ensure the blood clot’s stability in time [13]. On the contrary, other authors argue that the perforation of the sinus membrane does not affect the stability over time of the dental implants [14]. These studies show success rates from 100% to 95% of stability and osseointegration of the dental implants with this technique, but there is still no consensus on the minimal RBH in which they can be inserted safely [10,11,12,13,14].

Many authors also studied the endo-sinusal bone gain (ESBG) after performing the sinus lift technique with or without the use of bone grafts [15]. Randomized controlled clinical trials were performed in order to evaluate this aspect [15]. Their results indicated a significantly higher rate of ESBG in the case of sinus lifts with the use of bone grafts compared to no graft techniques [15]. However, the results obtained, although statistically significant, were carried out over a short period of time [15]. Other authors who studied this aspect in longer-term randomized clinical trials indicate a comparable ESBG in all patients regarding the technique used after more than 2 years post-surgery [16]. Similar to the study above, Qian et al. [12], in a study carried out over a period of 10 years, indicate no difference between groups regarding the ESBG at the end of the evaluated period.

The aim of this systematic review was to critically appraise the available evidence from clinical trials on the efficiency of sinus lift techniques with/without bone grafting in the atrophic posterior maxilla.

## 2. Materials and Methods

### 2.1. Participants, Intervention, Comparison, Outcome (PICO) Question

The protocol of this review was considered according to the Preferred Reporting Items for Systematic Review and Meta-Analyses (PRISMA) guidelines [17]. The focused question was elaborated according to the PICO question: “In patients with atrophic posterior maxilla (P), is there a difference in the efficiency of sinus lifts without bone graft (I) in comparison with sinus lifts and bone grafts (C) in terms of implant survival rate (ISR); marginal bone loss (MBL), ESBG (endo-sinus bone gain), and complications (O)?”

PICOS elements were considered as follows:

- Participants: adult patients with atrophic posterior maxilla in need of dental implants;
- Intervention: sinus lift without bone graft;
- Comparison: sinus with bone graft (e.g., autogenous, xenograft, allograft, alloplastic, biological agents of growth factors);
- Outcome: ISR (primary outcome); MBL, ESBG, biological complications, prosthetic complications (secondary outcome);
- Study type: randomized clinical trials (RCTs) or prospective controlled clinical trials (CCTs) with a follow-up ≥5 years.

### 2.2. Inclusion and Exclusion Criteria for RCTs

The inclusion criteria were as follows:

- Comparison sinus lifts without or with bone graft in the same RCT;
- Clinical trial with a follow-up ≥2 years;
- Fixed implants prosthodontic restored (cemented/screw-retained).

The exclusion criteria were as follows:

- In vitro, animal studies; non-RCT studies; systematic, narrative reviews, case reports, case series, monographs, letters to the editor;
- RCT with insufficient, missing, or unpublished data;
- RCT with a follow-up < 5 years;
- Articles published in other language than English.

### 2.3. Search Methods

Two reviewers (A.P.T., A.M.) performed an electronic search in PubMed, Scopus, and Web of Science databases until December 2023. To identify relevant clinical trials, the following keywords were used for electronic search: *sinus lift, sinus floor augmentation, sinus floor elevation, sinus membrane elevation, lateral approach sinus floor elevation, osteotome sinus floor elevation, crestal sinus floor elevation, transalveolar sinus floor elevation, bone graft, bone augmentation, guided bone regeneration, graftless, non-graft, and graft free*. In the first step, titles and abstracts were screened, and irrelevant trials were excluded. In the second step, after removing the duplicates, full-text studies previously obtained were examined and those who met the inclusion criteria were considered. If any disagreements were present, a third reviewer (F.O.) intervened with a resolution. A kappa analysis was conducted to assess the inter-rater reliability during this study selection process.

### 2.4. Data Extraction

The following data from the included trials were considered: first author, year of study, country, reference, type of RCT, patient characteristics and implants, type of sinus lift surgery, type of prosthetic restoration, primary outcome, secondary outcomes, and conclusions.

### 2.5. Risk of Bias

Cochrane Risk of Bias tool, version 2.0 [18], was considered for quality assessment of risk of bias. Seven domains were assessed for each RCT, including random sequence generation, allocation concealment, blinding of participants and/or personnel involved in this study, blinding of outcome assessment, incomplete outcome data reporting, selective reporting of outcomes, and other sources of bias. Each domain was analyzed by two reviewers (A.P.T., A.M.), and a third reviewer (F.O.) intervened if disagreements were present. A rating of low, unclear, or high risk of bias was performed for each domain.

### 2.6. Statistical Analysis

Statistical analysis was performed with RevMan (version 5.4, The Cochrane Collaboration 2020 [19], with a random effect model and a confidence interval (CI) of 95%. For the ISR, the risk ratio (RR) (CI 95%) was assessed using the Chi-Square Test [Mantel–Haenszel (M-H)]. For MBL and for ESBG, a weighted mean difference (WMD) (CI 95%) with sample size, inverse variance (IV), and standard error were assessed. The value of *p* < 0.05 was considered statistically significant. The heterogeneity among the trials was evaluated with an I-squared statistic test (*I*^2^), in which I^2^ values lower than 30% indicated low heterogeneity, between 30% and 60% indicated moderate heterogeneity and over 60% indicated substantial heterogeneity.

## 3. Results

### 3.1. Study Selection

**Figure 1:**
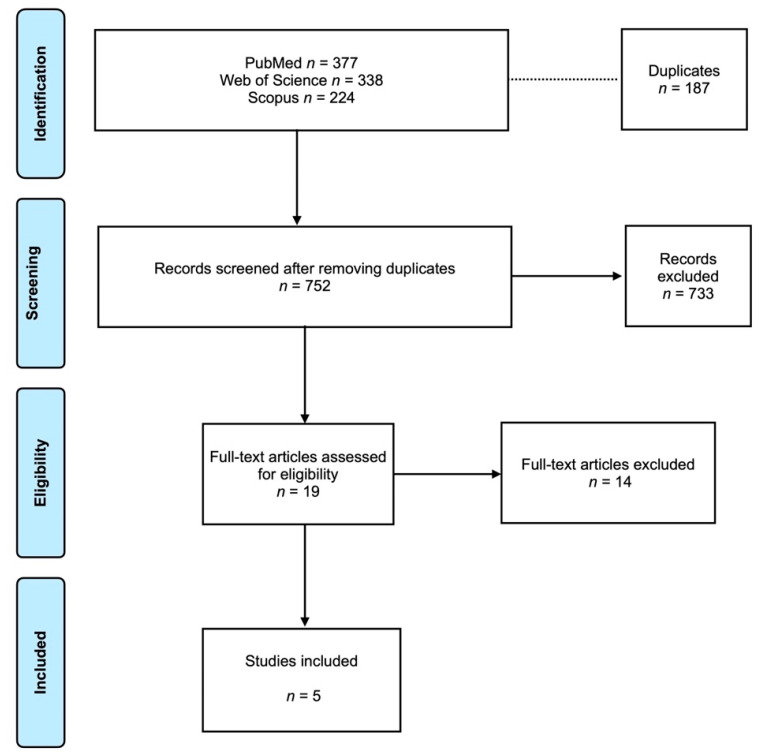
Prisma flowchart.

Figure 1 presents the Prisma flowchart. The electronic search retrieved 939 articles. After eliminating the duplicates (187 articles), 752 articles were screened. Finally, 19 articles were full-text assessed for eligibility. After the evaluation, 14 articles were excluded (the reason for exclusion is presented in Table 1). In the end, 5 RCTs were included [12,16,20,21,22]. The coefficient of Cohen’s kappa for inter-reviewer agreement was 0.96.

### 3.2. Study Description

The characteristics of each included study are detailed in Table 2. We included in our results five studies: two prospective RCTs [16,21], two RCTs [12,22], and one double-blind RCT [19]. The number of patients totally included was n = 175 [12,16,19,21]. The distribution of patients by sex was mentioned only in four studies [16,19,21] with a total of 60 males and 75 females. One study did not mention this aspect in their results, but the inclusion of 40 patients as a whole [15]. The mean age was between 48.5 and 56.7 years [16,22,23]. The majority of the studies used the OSFE technique [12,16,21,22,23], and one study used the lateral sinus lift approach [22]. A total of 380 implants were inserted simultaneously in each patient regarding the sinus floor elevation technique chosen [12,16,21,22,23]. From the total number of implants inserted, 59 implants are mentioned to be inserted simultaneously in the group of patients with no graft technique [12,19,20,21], while 64 implants are secondary to different bone grafting types [12,21,22,23]. Markovic et al. [16] and Ranaan et al. [22] did not mention the implant number distribution regarding the sinus floor elevation technique. The follow-up period of the cases was 2–10 years [12,16,21,22,23]. The implant survival rate was 94.1–100% secondary to no graft technique and 90–100% secondary to bone graft techniques [12,16,21,22,23]. In patients with no graft technique, the mean range of MBL in the final year of this study was reported as 0.6 ± 1.42 mm, while the ESBG was 3.07–4.8 mm [12,21,22,23]. Marcovic et al. [16] did not report any data regarding the MBL. Regarding the ESBG volume, Markovic et al. [16] report a range of 0.12–0.24 cc in the cases with grafting techniques and an ESBG of 0.22 cc in the no-grafting group. The most common biological complication was peri-implantitis [12,21,22,23]. The number of implants affected at the end of each study summed a total of 11 implants (no graft n = 6, graft n = 5) [12,21,22,23]. The most common prosthetic complications were ceramic/veneer chipping [12,16].

### 3.3. Risk of Bias Assessment

**Figure 2:**
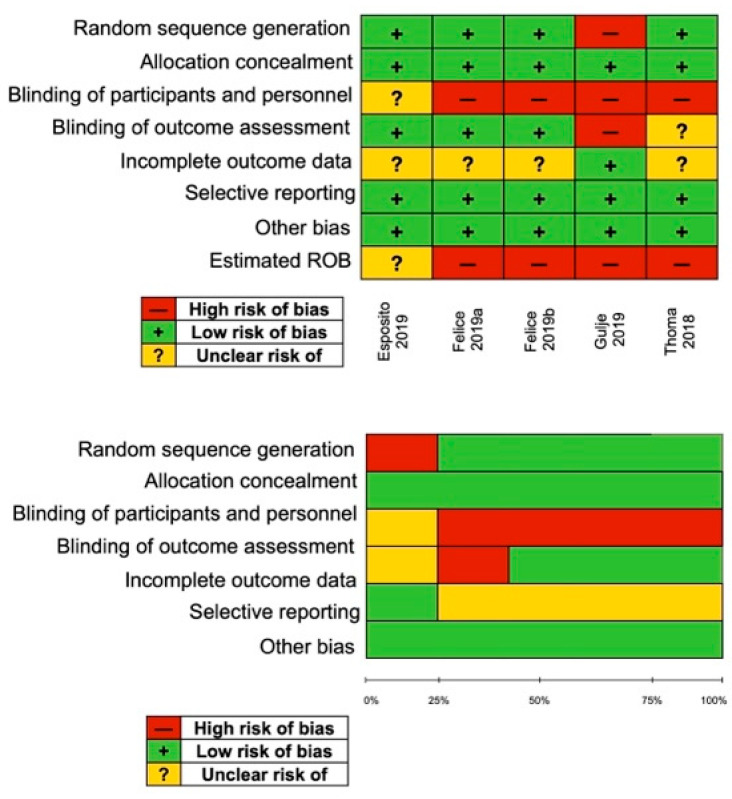
Cochrane ROB and GRADE tool assessment.

The results of the quality assessment are presented in Figure 2. Two RCTs were considered to have a low ROB, and three RCTs were considered with unclear ROB.

### 3.4. Statistical Analysis

**Figure 3:**
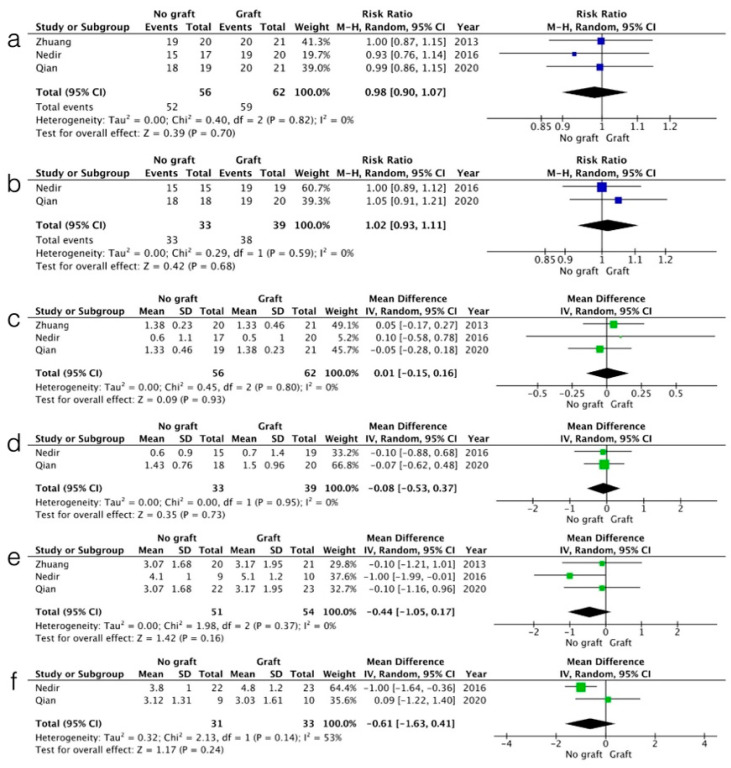
Statistical analysis for ISR at 3 years (**a**) and 5 years (**b**), MBL at 3 years (**c**) and 5 years (**d**), and ESBG at 3 years (**e**) and 5 years (**f**).

The ISR at 3 years had a RR of 0.98 [0.90, 1.07] (CI 95%), with I^2^ = 0% and *p* = 0.7 (Figure 3a), and at 5 years, RR was RR of 1.02 [0.93, 1.11] (CI 95%), with I^2^ = 0% and *p* = 0.68 (Figure 3b). The MBL at 3 years indicated a WMD of 0.01 [−0.15, 0.16] (CI 95%) with I^2^ = 0% and *p* = 0.93 (Figure 3c), and at 5 years, a WMD of −0.08 [−0.53, 0.37] (CI 95%) with I^2^ = 0% and *p* = 0.73 (Figure 3d). ESBG at 3 years had a WMD of −0.44 [−1.05, 0.17] (CI 95%) with I^2^ = 0% and *p* = 0.16 (Figure 3e), and at 5 years, WMD of −0.61 [−1.63, 0.41] (CI 95%) with I^2^ = 53% and *p* = 0.24 (Figure 3f).

## 4. Discussion

The aim of this study was to evaluate the effectiveness of sinus lift techniques with or without bone grafting. The low number of articles included in this systematic review is due to the fact that we aimed to include only those RCT articles that directly compared the success rate between the two methods of sinus lifting [13,20]. The low number of reports on this aspect is also confirmed by other previous meta-analyses [35,36]. Other authors included more articles in their research: Duan et al. [37] (22 studies), Moraschini et al. (18 studies) [38], and Starch-Jensen et al. (13 studies) [39]. However, in their limitation section, these authors have indicated an increased ROB from the high heterogeneity among the studies and because of the inclusion of several observational studies along with the RCTs [23,24,25].

In our study, we did not find any statistically significant difference between the two groups at 3 years (RR 0.98 (CI 95%, *p* = 0.07)), nor at a longer term of 5 years (RR of 1.02 (CI 95%, *p* = 0.68)). Similar results were reported by Alluden et al. [35] with an ISR of 95%, (CI = 0.90–0.99), Chen et al. [36]—ISR of 95%.—RR 1.012 (CI = 0.91, 1.120), Moraschini et al. [38]—ISR 95% CI 0.26, 1.19), *p* = 0.13, Duan et al. [37]—ISR = 97.9% (93–100%). In contrast, Starch-Jensen et al. [39] indicated in their meta-analyses a slight difference regarding the ISR in favor of the bone grafting surgical procedure: ISR = 96% non-grafted, 100% for bone-grafted patients. However, this study had only a short-term follow-up [39]. All authors support the fact that to ensure an optimal ISR in time, in the case of the no-graft technique, solid primary stability of the inserted implants is necessary [35,36,37,38,39,40].

Regarding the MBL in our study, there was no significant statistical difference between the groups. At 3 years we found a WMD of 0.01 [−0.15, 0.16] (CI 95%) *p* = 0.93, and at 5 years, WMD of −0.08 [−0.53, 0.37] (CI 95%) *p* = 0.73 regardless of the sinus floor elevation technique chosen (lateral or trans crestal approach). Our results are in line with the results of Chen et al. [36] and Duan et al. [37]. In contrast, other authors indicate in their results a correlation between RBH and MBL, with statistically significant results [35]. They found that MBL decreases in the case of implants inserted in patients with increased RBH, thus leading to an increase in ISR [20]. Alluden et al. [35] also report an increase in the ESBG index in edentulous patients with a higher RBH. Other studies reported also by Chen et al. [36] indicate a significant difference between the ESBG > 2 mm between the 2 groups [21,41]. Nedir et al. [21] report an ESBG > 2 mm at 5 years of 39.1% in the no-graft group of patients and a percent of 77.9% in the grafted group, while Pjetursson et al. [41] report an ESBG > 2 mm in 93.8% of the cases of non-grafted cases and 100% in the grafted ones.

The most frequent acute complication was the perforation of the sinus membrane, cases in which the surgical procedures were delayed until sinus membrane healing, and the patients were excluded from the studies. The most frequent late complication was the loss of dental implants, which in our study had an extremely low percentage. There were significant statistical differences between the two groups regarding this aspect, results that are in line with other meta-analyses performed [35,36,37,38,39,40]. Surgical complications and delayed wound healing with marginal peri-implantitis are favored by smoking, poor oral hygiene, or associated pathologies not controlled by the medication of the patients [35,36,37,38,39,40]. Some patients also complained of postoperative pain, a fact that we did not classify as a complication as such, being managed with an adjuvant anti-algesic treatment [21,22,23]. Additionally, this was in line with the results of other authors [35,36,37,38,39,40]. The most common prosthetic complication was ceramic/veneer chipping, and there were no differences as well between the two groups [35,36,37,38,39,40].

We found no statistical differences between the two groups regarding the analyzed variables, which suggests that the insertion of dental implants simultaneously with the no-graft sinus floor elevation technique can be approximately as safe as with the bone-grafted sinus floor elevation technique if the RHB ensures optimal primary stability in all of the cases. This is the first criterion of safe clinical practice. Primary stability can be achieved if the RHB has a measurable value on CBCT between 4 mm and 6 mm preoperatively [10,11,12,13]. Another criterion is that of the non-perforation of the sinus membrane during the surgical intervention. In this way, all conditions for preservation and isolation of the resulting blood clot will be ensured so that it can exercise all its characteristic osteoinductive and osteoconductive properties [13]. Precisely for these reasons, we and other authors believe that a good preservation of the sinus membrane can be done more easily in the future through the lateral approach, thus having direct visibility and additional safety for preserving its integrity [13].

However, this study has its limitations. The biggest of them all is due to the small number of articles included. To avoid this shortcoming in the future, we encourage and support the need to undertake more RCTs related to this aspect. Additionally, another major limitation is the complete absence of histopathological data. We consider that the confirmation of clinical and radiological results by histopathological analysis is absolutely necessary. The inclusion of the histopathological examination in future RCTs to support the initial results will contribute substantially to the development of surgical guidelines and protocols that are vitally needed.

## 5. Conclusions

The available evidence after the 30-year period analyzed in this review underlines that the osteotome sinus floor elevation technique without bone graft may be used when the residual bone height ensures optimal stability of the inserted dental implants.

## Figures and Tables

**Table 1:** Reason for articles exclusion [1,13,23,24,25,26,27,28,29,30,31,32,33,34].

Author	Reason
Shi et al. [23]	Comparison short vs. longer implants
Yue et al. [24]	Study protocol
Farina et al. [25]	No comparison graft vs. no graft
Pohl et al. [26]	Comparison short vs. longer implants
Zhou et al. [27]	No comparison graft vs. no graft
Gulje et al. [28]	Comparison short vs. longer implants
Bechara et al. [1]	Comparison short vs. longer implants
Merli et al. [29]	No comparison graft vs. no graft
Thoma et al. [30]	Comparison short vs. longer implants
Esposito et al. [31]	No comparison graft vs. no graft
Bruschi et al. [32]	Non-RCT study
Attia et al. [33]	Retrospective study
Pisoni et al. [34]	No comparison graft vs. no graft
Merheb et al. [13]	Same data from Nedir et al. [17]

**Table 2:** Characteristics of the included studies.

Author. Year. Country	Study Design	Patients Nr. Sex Age	Type of Sinus Lift	Type of Graft	Type of Implant	Follow-Up	Implant Survival Rate	Marginal Bone Loss (Mean)	ESBG	Prosthetic Type/Complications	Biologic Complication	Author Conclusion
Markovic 2015 Serbia. [16]	Prospective RCT	n = 45 Male n = 16 Female n = 29 Mean Age 56.7	OSFE	G1: No graft G2: Beta-TCP G3: DBB G4: Combined (DBB + TCP)	**All cases** n = 180 implants (45 implants/group) Length = 10 mm Diameter = 4.1 mm	2 yrs	100% All cases Lost n = 0	N/A	**Mean volume** **G1**—0.22 cc **G2**—0.24 cc **G3**—0.23 cc **G4**—0.12 cc	**Type** **Total** **n = 101** Bridges n = 79 Single crowns n = 22 **Complication** Ceramic chipping 3.9% Decementation or loosening of the crown 1.66%	N/A	The usage of grafting material offers **no significant advantage** to clinical success of dental implants
Zhuang. 2012 China [20]	Double-blind RCT	n = 45 Male n = 27 Female n = 18 Mean Age 48.5	OSFE	**G1:** DBB+ Autogenous bone chips harvested during drilling **G2:** No graft	**Total** n = 42 implants **G1** n = 21 implants Length = 6–10 mm **G2** n = 20 implants Length = 6–10 mm Diameter 4.1–4.8 mm	3 yrs	**G1** 95.2% Lost n = 1 **G2** 95% Lost n = 1	**G1** 1.33 ± 0.46 mm **G2** 1.38 ± 0.23 mm	**G1** **3 years** 3.17–1.95 mm **G2** **3 years** 3.07–1.68 mm	N/A	**Total** n = 2 **G1** N = 1 peri-implantitis **G2** n =1 peri-implantitis	The application of simultaneousgrafting **has no significant advantage** in terms of clinical success.
Nedir 2016 Switzerland [21]	Prospective RCT	n = 12 Male n = 3 Female n = 9 Mean Age N/A	OSFE	**G1:** No Graft **G2:** DBB	**Total** n = 37 implants G1: n = 17 implants Length = 8 mm G2: n = 20 implants Length = 8 mm	5 yrs	**G1** 94.1% Lost n = 2 **G2** 90% Lost n = 1	**G1** 0.6 ± 1.1 mm **G2** 0.6 ± 1.1 mm	**G1** **3 years** 4.1 mm **5 years** 3.8 mm **G2** **3 years** 5.1 mm **5 years** 4.8 mm	N/A	Total n = 3 G1 n = 1 peri-implatitis G2 n = 2 peri-implantitis	The new bone that Formed around implants in the first year was maintained at 5 years, **irrespective of the presence or the absence of grafting material.**
Qian 2019 China [12]	RCT	n = 40 Sex N/A Mean Age N/A	OSFE	**G1** DBB **G2** No graft	**Total** N = 40 **G1:** n = 21 Length 6–10 mm Diameter 4.1–4.8 mm **G2:** n = 19 Length 6–10 mm Diameter 4.1–4.8 mm	10 yrs	**G1**—90.7% Lost n = 2 **G2**—95.0% Lost n = 1	**G1** **3 years** 1.33 ± 0.46 **5 years** 1.50 ± 0.96 **10** **years** 1.67 ± 1.06 **G2** **3 years** 1.38 ± 0.23 **5 years** 1.43 ± 0.76 **10 years** 1.52 ± 1.08	**G1** **3 years** 3.17 ± 1.95 mm **5 years** 3.03 ± 1.61mm **10 years** 3.07 ± 1.34 mm **G2** **3 years** 3.07 ± 1.68 mm **5 years** 3.12 ± 1.31 mm **10** **Years** 3.14 ± 1.26 mm	**Type** **Total** **N = 40** Single crown **G1** N = 15 **G2** N = 14 Splinted implant **G1** = 6 **G2** = 5 **Complications per patient** **G1** Veneer Chipping n = 4 **G2** Veneer Chipping n = 3	**Total** n = 3 **G1** n = 1 peri-implantitis **G2** n = 2 periimplantitis	OSFE with or without grafting **both yielded predictable clinical outcomes.**
Ranaan 2018 USA [22]	RCT	n = 33 Male n = 14 Female n = 19 Mean age 57.42 years	Lateral	**G1** No graft **G2** Allograft	Total n = 76	2 yrs	94.76% G1 = G2 **G1** Lost n = 2 **G2** Lost n = 2	**G1** 0.85 (−0.38, −1.32) **G2**. 1.42 (−0.68, −2.16)	N/A	N/A	Total n = 4 G1 n = 2 peri-implantitis G2 n = 2 peri-implantitis	The stability of implants placed simultaneous with **Slit-window graft-free SFE was comparable to those placed simultaneous with lateral approach SFE in conjunction with bone grafting.**

## Data Availability

The original contributions presented in this study are included in the article. Further inquiries can be directed to the corresponding author.

## References

[1] Bechara S., Kubilius R., Veronesi G., Pires J.T., Shibli J.A., Mangano F.G. (2017). Short (6-mm) dental implants versus sinus floor elevation and placement of longer (≥10-mm) dental implants: A randomized controlled trial with a 3-year follow-up. Clin. Oral Implants Res..

[2] Pérez-Martínez S., Martorell-Calatayud L., Peñarrocha-Oltra D., García-Mira B., Peñarrocha-Diago M. (2015). Indirect sinus lift without bone graft material: Systematic review and meta-analysis. J. Clin. Exp. Dent..

[3] Valentini P. (2023). How to Prevent and Manage Postoperative Complications in Maxillary Sinus Augmentation Using the Lateral Approach: A Review. Int. J. Oral Maxillofac. Implants.

[4] Kim S.J., Ribeiro A.L., Atlas A.M., Saleh N., Royal J., Radvar M., Korostoff J. (2015). Resonance frequency analysis as a predictor of early implant failure in the partially edentulous posterior maxilla following immediate nonfunctional loading or delayed loading with single unit restorations. Clin. Oral Implants Res..

[5] Bathla S.C., Fry R.R., Majumdar K. (2018). Maxillary sinus augmentation. J. Indian. Soc. Periodontol..

[6] Al-Dajani M. (2016). Recent Trends in Sinus Lift Surgery and Their Clinical Implications. Clin. Implants Dent. Relat. Res..

[7] Amam M.A., Abdo A., Alnour A., Amam A., Jaafo M.H. (2023). Comparison of calcium sulfate and tricalcium phosphate in bone grafting after sinus lifting for dental implantation: A randomized controlled trial. Dent. Med. Probl..

[8] Einhorn T.A., Lee C.A. (2001). Bone regeneration: New findings and potential clinical applications. J. Am. Acad. Orthop. Surg..

[9] Laurencin C.T. (2003). Musculoskeletal allograft tissue banking and safety. Bone Graft Substitute.

[10] Moon J.W., Sohn D.S., Heo J.U., Shin H.I., Jung J.K. (2011). New bone formation in the maxillary sinus using peripheral venous blood alone. J. Oral Maxillofac. Surg..

[11] Rawat A., Thukral H., Jose A. (2019). Indirect Sinus Floor Elevation Technique with Simultaneous Implant Placement without Using Bone Grafts. Ann. Maxillofac. Surg..

[12] Qian S.J., Mo J.J., Si M.S., Qiao S.C., Shi J.Y., Lai H.C. (2020). Long-term outcomes of osteotome sinus floor elevation with or without bone grafting: The 10-year results of a randomized controlled trial. J. Clin. Periodontol..

[13] Merheb J., Nurdin N., Bischof M., Gimeno-Rico M., Quirynen M., Nedir R. (2019). Stability evaluation of implants placed in the atrophic maxilla using osteotome sinus floor elevation with and without bone grafting: A 5-year prospective study. Int. J. Oral Implantol..

[14] Ye M., Liu W., Cheng S., Yan L. (2021). Outcomes of implants placed after osteotome sinus floor elevation without bone grafts: A systematic review and meta-analysis of single-arm studies. Int. J. Implants Dent..

[15] Starch-Jensen T., Bruun N.H., Spin-Neto R. (2023). Endo-sinus bone gain following osteotome-mediated sinus floor elevation with Bio-Oss Collagen compared with no grafting material: A one-year single-blind randomized controlled trial. Int. J. Oral Maxillofac. Surg..

[16] Marković A., Mišić T., Calvo-Guirado J.L., Delgado-Ruíz R.A., Janjić B., Abboud M. (2016). Two-Center Prospective, Randomized, Clinical, and Radiographic Study Comparing Osteotome Sinus Floor Elevation with or without Bone Graft and Simultaneous Implant Placement. Clin. Implant. Dent. Relat. Res..

[17] Page M.J., McKenzie J.E., Bossuyt P.M., Boutron I., Hoffmann T.C., Mulrow C.D., Shamseer L., Tetzlaff J.M., Akl E.A., Brennan S.E. (2021). The PRISMA 2020 statement: An updated guideline for reporting systematic reviews. PLoS Med..

[18] Sterne J.A.C., Savović J., Page M.J., Elbers R.G., Blencowe N.S., Boutron I., Cates C.J., Cheng H.Y., Corbett M.S., Eldridge S.M. (2019). RoB 2: A revised tool for assessing risk of bias in randomised trials. BMJ.

[19] Deeks J.J., Higgins J.P.T., Altman D.G., McKenzie J.E., Veroniki A.A., Higgins J.P.T., Thomas J., Chandler J., Cumpston M., Li T., Page M.J., Welch V.A., on behalf of the Cochrane Statistical Methods Group (2019). Analysing data and undertaking meta-analyses. Cochrane Handbook for Systematic Reviews of Interventions.

[20] Si M.S., Zhuang L.F., Gu Y.X., Mo J.J., Qiao S.C., Lai H.C. (2013). Osteotome sinus floor elevation with or without grafting: A 3-year randomized controlled clinical trial. J. Clin. Periodontol..

[21] Nedir R., Nurdin N., Abi Najm S., El Hage M., Bischof M. (2017). Short implants placed with or without grafting into atrophic sinuses: The 5-year results of a prospective randomized controlled study. Clin. Oral Implants Res..

[22] Ranaan J., Bassir S.H., Andrada L., Shamshiri A.R., Maksoud M., Raanan R., Guze K. (2018). Clinical efficacy of the graft free slit-window sinus floor elevation procedure: A 2-year randomized controlled clinical trial. Clin. Oral Implants Res..

[23] Shi J.-Y., Lai Y.-R., Qian S.-J., Qiao S.-C., Tonetti M.S., Lai H.-C. (2021). Clinical, radiographic and economic evaluation of short-6-mm implants and longer implants combined with osteotome sinus floor elevation in moderately atrophic maxillae: A 3-year randomized clinical trial. J. Clin. Periodontol..

[24] Yue Z., Liu Q., Zhang H., Yang J., Hou J. (2021). Histological, radiological, and clinical outcomes of sinus floor elevation using a lateral approach for pre-/post-extraction of the severely compromised maxillary molars: A study protocol for a randomized controlled trial. Trials.

[25] Farina R., Riccardi O., Schincaglia G.P., Severi M., Trombelli L., Simonelli A. (2023). Six-year extension results of a randomized trial comparing transcrestal and lateral sinus floor elevation at sites with 3–6 mm of residual bone. Clin. Oral Implants Res..

[26] Pohl V., Thoma D.S., Sporniak-Tutak K., Garcia-Garcia A., Taylor T.D., Haas R., Hämmerle C.H. (2017). Short dental implants (6 mm) versus long dental implants (11–15 mm) in combination with sinus floor elevation procedures: 3-year results from a multicentre, randomized, controlled clinical trial. J. Clin. Periodontol..

[27] Zhou Y., Shi Y., Si M., Wu M., Xie Z. (2021). The comparative evaluation of transcrestal and lateral sinus floor elevation in sites with residual bone height ≤6 mm: A two-year prospective randomized study. Clin. Oral Implants Res..

[28] Guljé F.L., Raghoebar G.M., Gareb B., Vissink A., Meijer HJ A. (2024). Single crowns in the posterior maxilla supported by either 11-mm long implants with sinus floor augmentation or by 6-mm long implants: A 10-year randomized controlled trial. Clin. Oral Implants Res..

[29] Merli M., Moscatelli M., Mariotti G., Pagliaro U., Merli M., Nieri M. (2021). Use of autogenous bone versus deproteinised bovine bone matrix in one-stage lateral sinus floor elevation in severely atrophied maxillae: A 7-year randomised controlled trial. Int. J. Oral Implantol..

[30] Thoma D.S., Haas R., Sporniak-Tutak K., Garcia A., Taylor T.D., Hämmerle C.H.F. (2018). Randomized controlled multicentre study comparing short dental implants (6 mm) versus longer dental implants (11–15 mm) in combination with sinus floor elevation procedures: 5-Year data. J. Clin. Periodontol..

[31] Esposito M., Cannizzaro G., Barausse C., Cosci F., Soardi E., Felice P. (2014). Cosci versus Summers technique for crestal sinus lift: 3-year results from a randomised controlled trial. Eur. J. Oral Implantol..

[32] Bruschi G.B., Bruschi E., Papetti L. (2021). Flapless Localised Management of Sinus Floor (LMSF) for trans-crestal sinus floor augmentation and simultaneous implant placement. A retrospective non-randomized study: 5-year of follow-up. Heliyon.

[33] Attia S., Narberhaus C., Schaaf H., Streckbein P., Pons-Kühnemann J., Schmitt C., Neukam F.W., Howaldt H.P., Böttger S. (2020). Long-Term Influence of Platelet-Rich Plasma (PRP) on Dental Implants after Maxillary Augmentation: Retrospective Clinical and Radiological Outcomes of a Randomized Controlled Clinical Trial. J. Clin. Med..

[34] Pisoni L., Lucchi A., Persia M., Marchi O., Ordesi P., Siervo S. (2016). Sinus lift: 3 years follow up comparing autogenous bone block versus autogenous particulated grafts. J. Dent. Sci..

[35] Aludden H., Mordenfeld A., Hallman M., Christensen A.E., Starch-Jensen T. (2018). Osteotome-Mediated Sinus Floor Elevation with or without a Grafting Material: A Systematic Review and Meta-analysis of Long-term Studies (≥5-Years). Implant Dent..

[36] Chen M.H., Shi J.Y. (2018). Clinical and Radiological Outcomes of Implants in Osteotome Sinus Floor Elevation with and without Grafting: A Systematic Review and a Meta-Analysis. J. Prosthodont..

[37] Duan D.H., Fu J.H., Qi W., Du Y., Pan J., Wang H.L. (2017). Graft-Free Maxillary Sinus Floor Elevation: A Systematic Review and Meta-Analysis. J. Periodontol..

[38] Moraschini V., Uzeda M.G., Sartoretto S.C., Calasans-Maia M.D. (2017). Maxillary sinus floor elevation with simultaneous implant placement without grafting materials: A systematic review and meta-analysis. Int. J. Oral Maxillofac. Surg..

[39] Starch-Jensen T., Schou S. (2017). Maxillary Sinus Membrane Elevation With Simultaneous Installation of Implants Without the Use of a Graft Material: A Systematic Review. Implant. Dent..

[40] Lie S.A.N., Claessen R.M.M.A., Leung C.A.W., Merten H.A., Kessler P.A.W.H. (2022). Non-grafted versus grafted sinus lift procedures for implantation in the atrophic maxilla: A systematic review and meta-analysis of randomized controlled trials. Int. J. Oral Maxillofac. Surg..

[41] Pjetursson B.E., Rast C., Brägger U., Schmidlin K., Zwahlen M., Lang N.P. (2009). Maxillary sinus floor elevation using the (transalveolar) osteotome technique with or without grafting material. Part I: Implant survival and patients’ perception. Clin. Oral Implants Res..

